# Reliability and validity of the Edinburgh Postnatal Depression Scale (EPDS) for detecting perinatal common mental disorders (PCMDs) among women in low-and lower-middle-income countries: a systematic review

**DOI:** 10.1186/s12884-016-0859-2

**Published:** 2016-04-04

**Authors:** Sumitra Devi Shrestha, Rina Pradhan, Thach D. Tran, Rosa C. Gualano, Jane R. W. Fisher

**Affiliations:** Ministry of Health and Population, Kathmandu, Nepal; Life Line Nepal, Kathmandu, Nepal; Jean Hailes Research Unit, Monash University School of Public Health and Preventive Medicine, Level 1, 549 St Kilda Road, Melbourne, 3004 Victoria Australia; Nepal Institute of Health Sciences, Affiliated Purbanchal University, Kathmandu, Nepal

**Keywords:** Edinburgh postnatal depression scale (EPDS), Reliability, Validity, Cultural equivalence, Local language versions of the EPDS (LLV-EPDS), Perinatal common mental disorders (PCMDs), Low- and-lower-middle-income countries (LALMICs)

## Abstract

**Background:**

The Edinburgh Postnatal Depression Scale (EPDS), originally developed in Britain, is one of the most widely used screening instruments for assessing symptoms of the Perinatal Common Mental Disorders (PCMDs) of depression and anxiety. However, its potential to detect PCMDs in culturally diverse low- and lower-middle income countries (LALMICs) is unclear. This systematic review aimed to appraise formally validated local language versions of the EPDS from these resource-constrained settings.

**Methods:**

Following the PRISMA protocol, we searched MEDLINE-OVID, CINAHL-Plus and PUBMED to identify studies reporting translation, cultural adaptation and formal validation of the EPDS to detect PCMDs among women in LALMICs. The quality of the studies meeting inclusion criteria was assessed using standard criteria and a new process-based criteria; which was developed specifically for this study.

**Results:**

We identified 1281 records among which 16 met inclusion criteria; three further papers were identified by hand-searching reference lists. The publications reported findings from 12 LALMICs in14 native languages. Most of these local language versions of the EPDS (LLV-EPDS) had lower precision for identifying true cases of PCMDs among women in the general perinatal population compared to the original English version. Only one study met all criteria for culturally sensitive translation, the others had not established the comprehensibility of the local version amongst representative groups of women in pre-testing. Many studies tested the LLV-EPDS only amongst convenience samples recruited at single health facilities. Diagnostic interviews for confirmation of mental disorders could have been influenced by the mental health professionals’ lack of blinding to the initial screening results. Additionally, even when diagnostic-interviews were carried out in the local language, questions might not have been understood as most studies followed standard diagnostic protocol which had not been culturally adapted.

**Conclusions:**

Most of the LLV-EPDS from non-English speaking low- and middle-income-countries did not meet all criteria for formal validation of a screening instrument. Psychometric properties of LLV-EPDS could be enhanced by adopting the new process-based criteria for translation, adaptation and validation.

**Electronic supplementary material:**

The online version of this article (doi:10.1186/s12884-016-0859-2) contains supplementary material, which is available to authorized users.

## Background

There is growing recognition of mental health problems among women living in resource-constrained, World Bank defined low- and lower-middle-income countries (LALMICs) who are pregnant or have recently given birth. In order to address persistently high maternal and child morbidity and mortality and promote the survival, health and development of infants in these settings, national governments have expressed increasing interest in improving maternal mental health [1, 2]. To optimize detection of Perinatal Common Mental Disorders (PCMDs) among women in primary healthcare, locally adapted and validated screening instruments are needed.

The Edinburgh Postnatal Depression Scale (EPDS) is one of the most widely used screening instruments for assessing symptoms of perinatal depression and anxiety [3, 4]. It assesses emotional experiences over the past seven days using ten Likert-scale items (See Additional file 1). This self-reporting instrument was originally developed in the United Kingdom (U.K.) by Cox, Holden and Sagovsky in 1987 [5]. Its use has now extended far beyond the U.K. to other high-income English-speaking and non-English speaking countries, and increasingly to non-Anglophone LALMICs. The popularity of this brief instrument reflects the original British validation study [6], in which nine out of ten women who were diagnosed by a psychiatrist as being depressed after giving birth were correctly identified in a blinded comparison with scores above a cut-off on the EPDS. The psychometric properties of the EPDS in primary health care were: 86 % sensitivity (correctly identifying true cases), 78 % specificity (correctly identifying people without the condition) and 73 % positive predictive value (proportion of respondents scoring positive in the test who had a mental disorder diagnosed by clinical interview) [6].

To improve early detection and treatment of PCMDs, the local language versions of the EPDS (LLV-EPDS) needs to accurately identify people with a PCMDs. However, it has been found that the LLV-EPDS had relatively lower discriminant validity for correctly identifying cases of PCMDs [3, 4] than the original English version [6]. Many reasons have been proposed for why LLV-EPDS did not perform well. These include lack of local cultural sensitivity [3, 7–9] due to compromises made during translation and adaptation process [10] and recruitment of participants who did not represent general perinatal populations [4]. Finally, the questions asked during diagnostic interviews (the standard comparator) might not have been meaningful or comprehensible in these local settings. Development of a LLV-EPDS with optimal psychometric properties is fundamental for identification of perinatal mental disorders, for assisting nations to assess the overall burden of PCMDs [11] and enabling aggregation of global prevalence data [10]. However, there is no internationally approved, standard technique for translation and validation of the English EPDS into a non-English local language version appropriate for use in resource-constrained countries.

There are three systematic reviews on the validity of non-English versions of the EPDS. Two of these studies were focused on publications from high and middle-income countries, and included few studies from low-income countries [3, 4], while the other review included only data from African countries [12]. There is no systematic review specific to low-and lower-middle-income countries (LALMICs) [13]. The objectives of this review were: (1) to appraise systematically the formally validated LLV-EPDS from LALMICs, and (2) to establish potentially modifiable reasons for their lower validity by using new specific process-based criteria.

## Methods

### Search strategy

We used the Preferred Reporting Items for Systematic reviews and Meta-Analyses (PRISMA) protocol for identifying, screening and eligibility of studies [14] (Additional file 2). Three indexed electronic international databases (MEDLINE-OVID, CINAHL-Plus and PUBMED) were searched up to 20 April 2015, using the strategy described in Additional file 3.

### Inclusion criteria

There were four inclusion criteria: studies on translation and/or cultural adaptation and/or validation of the EPDS; that enrolled women who were pregnant and/or had recently given birth; which were conducted in World Bank defined LALMICs, and with reports published in English language, peer-reviewed journals.

### Selection of studies

In addition to implementing the search strategy, the reference lists of articles meeting inclusion criteria were searched to identify any studies that had not been found. In order to obtain copies of studies published in non-indexed or local journals, we corresponded with authors via email, if we did not receive a response we wrote to the editor of the journal. By learning the journal was no more published, we sought a copy of the publications through interlibrary loan.

### Quality assessment

We used two approaches to assess the methodological quality of the selected publications. First, overall quality was assessed using the criteria recommended by Mirza and Jenkins [15]. As recommended by Fisher et al. [1] we added a criterion about whether approval from a formally constituted ethics committee had been obtained. Thus, the 9 criteria were: (1) clear study aim; (2) sufficient sample size or justification; (3) representativeness of the sample or justification; (4) explicit inclusion and exclusion criteria; (5) response rate and explanation of losses; (6) clear description of data; (7) appropriate statistical analyses; (8) ethics approval; (9) and obtained informed consent. One point was given for meeting each of these points (1for Yes and 0 for No), to yield a maximum total possible score of 9.

**Fig. 1:**
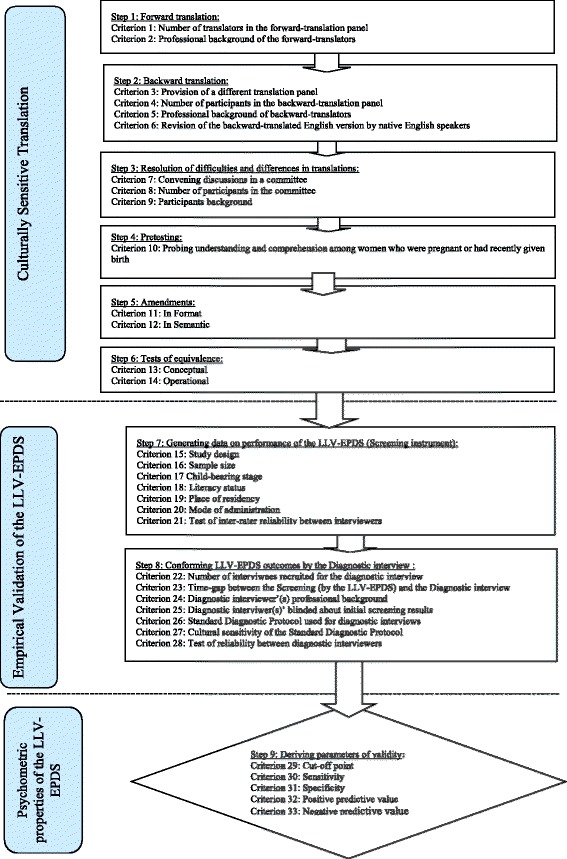
Development of Process-based review criteria for assessing formally validated local language versions of the EPDS (LLV-EPDS) in low- and lower-middle-income countries

The Mirza & Jenkins [15] and Fisher et al. [1] assessment scheme did not include specific criteria for assessing quality of a screening instrument like the EPDS. We assessed quality of the translation, cultural adaptation and local validation of the LLV-EPDS by developing a new set of process-based criteria (shown in Fig. 1, and defined in Additional file 4). We derived 33 criteria from diverse sources: which were recommended points for translation of other psychometric instruments [10, 16] and self-reporting questionnaires (SRQ) [17], measures used and suggested for translation and validation of the EPDS by earlier studies [3, 7, 11, 18–22]. Additionally, we incorporated some criteria based on our experience in international public health.

### Collection of data and analysis

We extracted data from selected studies using data-extraction format (Table 1).

**Table 1:** Studies included for the systematic review on reliability and validity of the Edinburgh Postnatal Depression Scale in low- and lower-middle-income countries

Authors/Year	Setting	Study type	Sample and recruitment	Outcome Variable	Outcome Measured	Psychometric properties of the LLV-EPDS
1. Nepal et al., (1999) [24]	Specialised hospital for women and university teaching hospital, located in the Kathmandu (capital), Nepal.	Cross-sectional	• 132/149 postnatal women • Recruited from the maternity wards of respective hospitals (≥2 day’s post-delivery) and followed up after 4 weeks.	Psychometric properties	Cut-off point; Sensitivity (Se); Specificity (Sp); Positive predictive value (PPV); Negative predictive value (NPV)	Nepalese version • Cut-off point: 12/13 • Se: 68.4 • Sp: 93.8 • PPV: 65 • NPV: 94.6
2. Regmi et al., (2002) [40]	University teaching hospital, located in the Kathmandu (capital), Nepal.	Cross-sectional	• 100 postnatal women (2–3 months) • Recruited from the post-natal/child immunization clinic. It recruited 40 non-child bearing women as controls (*mainly nurses & their friends*).	Psychometric properties	Cut-off point; Sensitivity (Se); Specificity (Sp); Positive predictive value (PPV); Negative predictive value (NPV)	Nepalese version • Cut-off point: 12/13 • Se: 100 • Sp: 92.6 • PPV: 41.6 • NPV: 100
3. Patel et al., (2002) [28]	Hospital in the Northern town of Goa (Western State), India.	Prospective	• 270/297 pregnant women (≥30 week) • Recruited from the antenatal clinics, and then followed 6–8 weeks after delivery.	Psychometric properties PCMDs prevalence	- Cut-off point; Sensitivity (Se); Specificity (Sp) - PCMDs prevalence	Konkani version: • Cut-off point: 11/12 • Se: 92 • Sp: 85
4. Uwakwe (2003) [30]	University teaching hospital, located in the Eastern State, Nigeria.	Cross-sectional	• 225/292 postnatal women • Recruited from the maternity ward (≥7 day’s post-delivery) & then followed in the first postnatal clinic visit.	Psychometric properties	Cut-off point; Sensitivity (Se); Specificity (Sp); Positive predictive value (PPV); Negative predictive value (NPV)	Igbo version (Eastern) • Cut-off point: 8/9 • Se: 75 • Sp: 97 • PPV: 75 • NPV: 97
5. Fisher et al., (2004) [23]	Maternal and Child and Family Planning Centre, located in the Ho Chi Minh city, Viet Nam.	Cross-sectional	• Cross-sectional, 506 postnatal women (6–8 weeks) • Recruited from the infant health clinics or those came for medical review	PCMDs prevalence	PCMDs prevalence	Reason included for this Review Earlier Vietnamese version-EPDS (developed by Small et al., (1999) [43] for women living in Australia) was revised before assessment of prevalence
6. Rahman et al., (2005) [39]	Rural sub-district of Rawalpindi, Pakistan.	Cross-sectional	• 541/570 postnatal women (10 to12 weeks) • Recruited from community.	Psychometric properties	Cut-off point; Sensitivity (Se); Specificity (Sp); Positive predictive value (PPV)	Urdu version • Cut-off point: 9/10^a^ • Se: 81.5 • Sp: 73.5 • PPV: 52.6
7. Adewuya et al., (2006) [31]	5 Health centres, located in a semi-urban town of the Western Nigeria.	Cross-sectional	182 pregnant women (≥32 weeks) • Recruited from antenatal clinics.	Psychometric properties	Cut-off point; Sensitivity (Se); Specificity (Sp); Positive predictive value (PPV); Negative predictive value (NPV)	Yoruba version (Western) • Cut-off point: 9/10 • Se: 86.7 • Sp: 91.5 • PPV: 68.4 • NPV: 97
8. Pollock et al., (2006) [32]	Central Psychiatric Hospital, Mental Health and Necrology Centre, and 3 Primary Health Care Centres (PHCCs), located in the Ulaanbaatar (capital), Mongolia.	Cross-sectional	• 94/100 women (in reproductive age) - Recruited from psychiatric units (55) - Rest from PHCCs immunization clinics	Psychometric properties	Cut-off point; Sensitivity (Se); Specificity (Sp); Positive predictive value (PPV); Negative predictive value (NPV)	Mongolian version • Cut-off point: 12/13 • Se: 80.9 • Sp: 61.7 • PPV: 67.9 • NPV: 76.3
9. Gausia et al., (2007) [19]	Social and Behavioural Sciences Unit (SBSU) and Hospital, located in the Dhaka (capital), Bangladesh.	Cross-sectional	• 10 female employee from SBSU • 11 mothers (baby ≤ 1 year) attending immunization clinic • 4 women whose infants were admitted to the hospital	Cultural and operational equivalence of Bangla version EPDS	Correlation between Bangla and original English version Correlation between self-report and interview administration	• Bangla and original English version (0.981; *p* < 0.01) • Self-report and interview ((0.752; *p* = 0.01)
10. Gausia et al., (2007) [25]	Hospital, located in the Dhaka (capital), Bangladesh.	Cross-sectional	• 100 /126 postnatal women (6–8 weeks) • Recruited from child immunization clinic.	Psychometric properties	Cut-off point; Sensitivity (Se); Specificity (Sp); Positive predictive value (PPV); Negative predictive value (NPV)	Bangla version • Cut-off point: 9/10 • Se: 88.9 • Sp: 86.8 • PPV: 40 • NPV: 98.6
11. Rowel et al., (2008) [37]	Field polyclinics in Kolonnawa, Western part of the Colombo (capital), Sri Lanka.	Cross-sectional	• 465 perinatal women recruited: - 265 pregnant (≥34 weeks) attending antenatal clinics. - 204 postpartum women (≥6 weeks) attending family planning or child wellbeing clinics.	Psychometric properties	Cut-off point; Sensitivity (Se); Specificity (Sp)	Sinhalese version • Cut-off point: 8/9 Pregnant • Se: 90.7 • Sp: 86.8 Postnatal • Se: 89.9 • Sp: 78.9
12. Hanlon et al., (2008) [33]	Butajra (rural region) located 130 km South of the Addis Abba (capital), Ethiopia.	Cross-sectional	• 101 postnatal women (median 5 Months) • Recruited from the community.	Psychometric properties	Cut-off point; Sensitivity (Se); Specificity (Sp)	Amharic version 1. Cut-off point: 5/6 • Sp: 76.5 • Se: 36.1
13. Weobong et al., (2009) [38]	Brong-Ahafo region (South part), Ghana.	Cross-sectional	• 160 pregnant women (5–11 week) • Identified from the database of 1/6 districts where vitamin A trial initiated.	Psychometric properties	Cut-off point; Sensitivity (Se); Specificity (Sp); Positive predictive value (PPV); Negative predictive value (NPV)	Twi version • Cut-off point: 10/11 • Se: 78 • Sp: 73 • PPV: 22 • NPV: 97
14. Tesfaye et al., (2010) [35]	2 Primary Health Care Centres, located in peri-urban area of the Addis Ababa (capital), Nigeria.	Cross-sectional	• 100/102 postnatal women (6 to 14 weeks) • Recruited from child immunization and postnatal clinics.	Psychometric properties	Cut-off point; Sensitivity (Se); Specificity (Sp); Positive predictive value (PPV); Negative predictive value (NPV)	Amharic version • Cut-off point: 6/7 • Se: 78.9 • Sp: 75.3 • PPV: 42.9 • NPV: 93.8
15. Chibanda et al., (2010) [36]	2 Primary Health Care Centres located in peri-urban area of the Harare (capital), Zimbabwe.	Cross-sectional	• 210/223 postnatal women (6–7 weeks) • Identified by computer generated randomization of clinic review cards, • Recruited from the 2 primary health care centres	Psychometric properties	Cut-off point; Sensitivity (Se); Specificity (Sp); Positive predictive value (PPV); Negative predictive value (NPV)	Shona version • Cut-off point: 10/11^a^ • Se: 88 • Sp: 87 • PPV: 74 NPV: 94
16. Fernandes et al. (2010) [29]	Missionary hospital located in rural part of Karnataka State (South), India.	Cross-sectional	• 194/196 pregnant (32 – 38 weeks) women • Recruited from the antenatal clinic.	Psychometric properties	Cut-off point; Sensitivity (Se); Specificity (Sp); Positive predictive value (PPV); Negative predictive value (NPV)	Kannada version • Cut-off point: 12/13 • Se: 100 • Sp: 84.9 • PPV: 52 • NPV: 99
17. Tran et al., (2011) [27]	Randomly selected Commune Health Centres (CMCs) from the Hanoi (capital) and Ha Nam province, Vietnam.	Cross-sectional	• 364/392 perinatal women - 199 were ≥ 28 weeks pregnant - Rest were 4–6 weeks postpartum) • Mostly were recruited from the CMCs and in Ha Nam province, also house visit for some.	Psychometric properties	Cut-off point; Sensitivity (Se); Specificity (Sp); Positive predictive value (PPV); Negative predictive value (NPV)	Vietnamese version • Cut-off point: 3/4^a^ • Se: 69.9 • Sp: 72.9 • PPV: 69.7 • NPV: 72
18. Husain et al., (2013) [26]	An urban slum in the Karachi (capital), Pakistan.	Cross-sectional	• 601/664 postnatal women (0–36 months) • Recruited from the slum.	Psychometric properties	Cut-off point; Sensitivity (Se); Specificity (Sp); Positive predictive value (PPV); Negative predictive value (NPV)	Urdu version • Cut-off point: 13/14^a^ • Se: 79 • Sp: 74 • PPV: 82 • NPV: 70
19. Stewart et al., (2013) [34]	District hospital, Mangochi, (Southern township), Malawi.	Cross-sectional	• 224 pregnant women (2nd trimester) • Recruited from antenatal clinic.	Psychometric properties	Cut-off point; Sensitivity (Se); Specificity (Sp); Positive predictive value (PPV); Negative predictive value (NPV)	Chichewa version • Cut-off point: 4/5^a^ • Se: 68.7 • Sp: 88.2 • PPV: 35.8 • NPV: 97.4

^a^Of the multiple cut-offs: presented one that had Sensitivity and Specificity nearest to 80 %

Adherence to the 33 process-based criteria were organised into two sections: Culturally Sensitive Translation and Empirical Validation using three response options: Yes/Not mentioned/Not needed (Tables 3 and 4). In line to aim of this study, data (evidence) about adherence to the process-based criteria summarised as narratives by three key aspects of LLV-EPDS development process: Culturally Sensitive Translation, Empirical Validation and Psychometric Properties (meta-analysis was not done as was beyond our study objectives). The data on process-based criteria and methodological quality were extracted by the first author and then rechecked by other authors; differences were resolved by consensus.

## Results

**Fig. 2:**
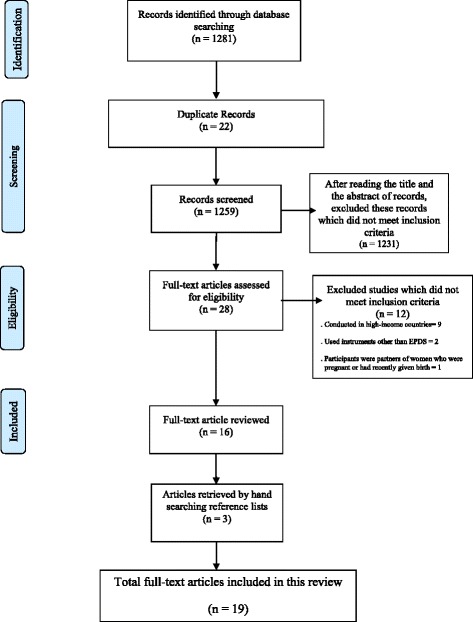
Selection of studies for review on cultural adaptation and validation of the EPDS in low- and lower-middle-income countries (defined as per World Bank criteria)

In total 1281 records were identified using the search strategy, after removal of duplicates and studies which did not meet inclusion criteria, including six articles published in languages other than English (French, Lithuanian, Polish, Turkish, Hebrew and German), but not conducted in LALMICs,19 studies, all quantitative, remained (Fig. 2 and Table 1).

Methodological assessment of these 19 selected publications indicated that five studies had enrolled sufficient participants to achieve adequate power. Overall, all had a clearly stated aim, 16 studies used clearly defined criteria for selection of the participants. Nine reported representativeness of samples with justification, 12 provided the recruitment rate, 14 publications included a synopsis of participants’ characteristics, 14 acquired ethical approvals, three studies described how participants’ consent had been acquired, but the remaining studies did not. Almost all studies had used appropriate statistical analyses for deriving psychometric properties (Table 2). All studies were included in this review despite of their modest quality. Since, this study aimed to identify reasons behind formally validated LLV-EPDS having lower validity than the original English version.

**Table 2:** Methodological quality of studies on translation and validation of the Edinburgh Postnatal Depression Scale in low- and lower-middle-income countries

Study	Clear study aim	Sample adequacy (justification)	Representative sample (with justification)	Explicit criteria for inclusion & exclusion	Response rate	Description of data	Appropriate statistical analyses	Ethics approval	Obtained informed consent from participants	Total score
1. Nepal et al., (2002) [24]	1	0	0	1	1	1	0	0	0	4
2. Regmi et al., (2002) [40]	1	0	0	1	1	0	1	1	0	5
3. Patel et al., (2002) [28]	1	0	0	1	1	1	1	0	1	6
4. Uwakwe (2003) [30]	1	0	0	1	1	1	1	0	0	5
5. Fisher et al., (2004) [23]	1	1	1	1	1	1	1	1	0	8
6. Rahman et al., (2005) [39]	1	1	1	1	1	1	1	1	0	8
7. Adewuya et al., (2006) [31]	1	0	0	1	0	1	1	1	0	5
8. Pollock et al., (2006) [32]	1	0	0	1	1	0	1	1	1	6
9. Gausia et al., (2007) [19]	1	0	1	1	1	1	1	1	0	7
10. Gausia et al., (2007) [25]	1	0	1	1	0	1	1	1	0	6
11. Rowel et al., (2008) [37]	1	1	1	1	0	0	1	0	0	5
12. Hanlon et al., (2008) [33]	1	0	1	0	1	1	1	1	0	6
13. Weobong et al., (2009) [38]	1	0	1	0	0	1	1	0	0	4
14. Tesfaye et al., (2010) [35]	1	0	1	0	1	1	1	1	0	6
15. Chibanda et al., (2010) [36]	1	0	0	1	1	1	1	1	1	7
16. Fernandes et al. (2010) [29]	1	0	0	1	0	0	1	1	0	4
17. Tran et al., (2011) [27]	1	1	1	1	0	1	1	1	0	7
18. Husain et al., (2013) [26]	1	1	0	1	1	0	1	1	0	6
19. Stewart et al., (2013) [34]	1	0	0	1	0	1	1	1	0	5

Of these 19 publications, two studies described translations of the original English EPDS into local languages, one entirely on the topic and another dealt briefly [19, 23]; 13 studies discussed both translation and psychometric properties of the LLV-EPDS; and four studies [24–27] focused on establishing psychometric properties for LLV-EPDS that were already translated. In total 17 studies described psychometric properties of the LLV-EPDS by recruiting a total of 4029 women (sample sizes ranged from 100 to 601) from 12 LALMICs [24–40].

### Culturally Sensitive Translation

We found 15 publications reporting translations and adaptation of 14 LLV-EPDS from 12 countries; there were two studies from each of three countries. India [28, 29] and Nigeria [30, 31] each had two native language versions of the EPDS. Both studies from Ethiopia [33, 35] were related to the development of an Amharic version of the EPDS.

In these 15 studies there was variable application of the six key steps we proposed for the culturally sensitive translation of the EPDS: forward translation, backward translation, resolution of difficulties and differences in translations by committee approach, pretesting, amendments and test of conceptual and operational equivalence (Table 3). Seven studies described the translation process and reported forward and backward translation by native speakers who were fluent in English as well. There was a predominance of health professionals in translation panels, which typically had 1–4 members. Two forward-translation panels [19, 32] had included professional translators. In eight studies there were separate panels [19, 24, 29–34] for forward and backward translations. Only one study reported review of the back-translated version by native English speakers [19].

**Table 3:** Culturally Sensitive Translation of the Edinburgh Postnatal Depression Scale in 12 low -and lower-middle-income countries

Country and Author	Forward translation	Back translation	Resolution of differences in translations by Committee approach	Pretesting	Amendments	Test of Equivalence	Translated version
1. Nepal							
Nepal et al., (1999) [24]	Yes (psychiatrist)	Yes (Another set)	NM	Yes	Not needed	NM	Nepalese
2. India							
Patel et al., (2002) [28]	Yes	Yes	NM	NM	NM	NM	Konkani
Fernandes et al. (2010) [29]	Yes (1 health professional)	Yes (Another set: health professional)	Yes (investigator, translators & another member of the study team)	Yes	NM	NM	Kannada
3. Nigeria							
Uwakwe (2003) [30]	Yes (3 nurses)	Yes (Another set: 2 medical students & layperson)	Yes (investigator&translators)	Yes	Not needed	NM	Igbo (Eastern)
Adewuya et al., (2006) [31]	Yes (Psychiatrist & linguist)	Yes (Another set: psychiatrist & linguist)	NM	NM	NM	NM	Yoruba (Western)
4. Pakistan							
Rahman et al., (2005) [39]	Yes	Yes	\Yes	NM	NM	NM	Urdu
5. Mongolia							
Pollock et al., (2006) [32]	Yes (2 medical translators)	Yes (Another set: 2 medical translators)	Yes (investigator, psychiatrist & doctor)	NM	Yes	NM	Mongolian
6. Bangladesh							
Gausia et al., (2007) [19]	Yes (1 Principal investigator)	Yes (Another set: translator & 2 native English speakers)	Yes (investigator,1 psychologist, 1 psychiatrist, 1 paediatrician, 3 physicians, 2 lay persons)	Yes (Probing)	Yes	Yes	Bengali
7. Sri Lanka							
Rowel et al., (2008) [37]	Yes	Yes	NM	Yes	NM	NM	Sinhalese
8. Ethiopia							
Hanlon et al., (2008) [33]	Yes (Physicians) Yes (Another set: physicians)		Yes (investigator,2 senior psychiatrists)	Yes (Probing)	Yes	NM	Amharic
Tesfaye et al., (2010) [35]	*Re-validation of the earlier EPDS –Amharic version (Hanlon* et al.*,* [33]*)*		NM	Yes (Probing)	Yes	NM	
9. Ghana							
Weobong et al., (2009) [38]	Yes (Native & UK professionals)	Yes (Native & UK professionals)	Yes (study team)	(Qualitative study)	Not needed	NM	Twi
10. Zimbabwe							
Chibanda et al., (2010) [36]	Yes (research assistant)	Yes	Yes (study team)	NM	NM	NM	Shona
11. Vietnam							
‡Fisher et al., (2004) [23]	Yes	Yes	Yes (study team)	Yes (Probing)	Yes	NM	Vietnamese^a^
12. Malawi							
Stewart et al., (2013) [34]	Yes (1 health professional, psychiatrist (UK), 2 social science graduates)	Yes (Another set: 1 non-mental health professional)	Yes (antenatal clinic nurses)	Yes	Yes	NM	Chichewa (^b^Visual Prompt card

*NM* not mentioned; ^a^Fisher et al. [23] revised Vietnamese version developed by Small et al. (1999) [43]; ^b^Visual Prompt card depicting a range of happy and sad faces was used along with the Chichewa-version-EPDS

Nine studies resolved differences in translation by convening a large group discussion; however in most of these studies (8/9), such discussions were held amongst diverse health professionals and in two studies professional translators were also included [29, 30]. Only one study used a committee approach, which included lay-people in addition to multi-disciplinary health professionals, and there were a number of meetings during the translation process [19].

Nine studies reported pre-testing the preliminary versions of the LLV-EPDS, however only four of these studies (involving the Amharic version from Ethiopia, along with the Bangla and Vietnamese versions) [19, 23, 33, 35] probed understanding and comprehension among women who had recently given birth. One type of amendment in these three LLV-EPDS translations was in the format of the instrument. For example, in the Bangla version to enable administration by an interviewer for respondents who were illiterate, women were addressed in the second person (‘you’). To reduce repetition, the short reply statements were replaced by numerals [19]. In the Amharic version, all ten items were changed into a question. The mode of response was changed into two stages: first by asking a fixed choice Yes/No question, then in the next step probing the frequency and severity of the reported symptoms. Additionally, the short reply statements were rephrased for clarity. Still more, to remind study participants about the timeframe the phrase “last week” was added at the end of the each item [33].

The other type of amendment aimed to establish semantic equivalence of the LLV-EPDS in the local context. Altogether four studies [19, 23, 33, 35] altered 8/10 EPDS items in this manner. Among three LLV-EPDS (Amharic, Bangla and Vietnamese) versions, we found an overlap among items modified in the different versions, but there were also items that were modified in one study but not in others. In a study conducted in Ethiopia, Hanlon et al. [33] modified six items in the Amharic version (1–5 & 9), and to ease understanding by rural women including illustrative examples for items 1–3. Despite these modifications, in a re-validation of this version with urban-dwelling women who had recently given birth, Tesfaye et al. [35] found that items 1 and 2 were still not understood and that provision of examples did not help respondents to understand these question. Connotative translations using local expression were reported for item 6 for the Vietnamese [23] and the Bangla [19] versions. Similarly, item 10 (relating to suicidal ideation) was rephrased in the Vietnamese version as the content was ambiguous in translation [23]. In Ethiopia mixed responses were found; women in rural areas were open to the question about ideas of self harm [33], while women in urban areas without this symptom were embarrassed to be asked it, but women who reported had suicidal ideas reported relief at the interviewer asking this question [35].

The tests of conceptual and operational equivalence of these LLV-EPDS with the original English version was performed only for the Bangla version EPDS. The test showed a higher conceptual equivalence, with the original English EPDS (correlation coefficient 0.981; *p* < 0.01). The operational equivalence test, which is to investigate whether the local version can achieve similar outcomes when administered by self-report as by an interviewer was slightly lower (correlation coefficient 0.752; *p* = 0.01) [19].

### Empirical Validation

There were 17 studies on psychometric validations of 14 LLV-EPDS from 12 countries; due to inclusion of two studies from each of five countries: Ethiopia [33, 35], India [28, 29], Nepal [24, 40], Nigeria [30, 31] and Pakistan [26, 39] (Table 4).

**Table 4:** Empirical Validation of the local language versions of the Edinburgh Postnatal Depression Scale in 12 low- and- lower-middle-income countries

Country and Author	Participant characteristics	Mode of administration	Sub-sample /Sample	Same day	Blinded	Administered by	Diagnostic Instruments^c^ & diagnostic criteria^d^	Cut-off	Se^e^ (%)	Sp^f^ (%)	PPV^g^ (%)	NPV^h^ (%)
Nepal												
Nepal et al., (1999) [24]	132/149 postnatal women convenientlyrecruited from the maternity wards of two hospitals (≥2 days post-delivery) located in the Kathmandu. Then followed up after 4 weeks (84.5 % were literate).	Interview	All 132 participants	NM	NM	2 Psychiatrists	DSM-IV of major depression	12/13	68.4	93.8	65	94.6
^a^Regmi et al., (2002) [40]	100 postnatal women (2–3 months) conveniently recruited from the post-natal clinic of the university teaching hospital in the Kathmandu. This case-controlled study recruited40 non-child bearing women as controls (*mainly nurses & their friends*).	Self-reporting	30 postpartum women (all 12 scored ≥ 13 and rest scored ≤12 were randomly selected)	NM	NM	NM	SCID DSM-IV of major depression		100	92.6	41.6	100
India												
Patel et al., (2002) [28]	270/297 pregnant (≥30 week) women conveniently recruited from antenatal clinics, and then followed 6–8 weeks after delivery (252), in Goa. Konkani, Marathi, Hindi and English speakers were included for this study. In this state, the female literacy rate is 67 % and 87 % of births are supervised.	Interviewers	Not clear	NM	NM	NM	CIS-R of common mental disorders	11/12	92	85	NM	NM
Fernandes et al. (2010) [29]	194/196 pregnant (32 – 38 weeks) women conveniently recruited from the antenatal clinic of the missionary hospital located in the rural area of Karnataka state. 95.5 % of these women had completed primary education.	Interviewer	All 194	Yes	NM	1 psychologist	MINI DSM-IV of depression *(translated)*	12/13	100	84.9	52	99
Nigeria												
Uwakwe (2003) [30]	225/292 postnatal women conveniently recruited from the maternity ward (≥7 days post-delivery), of a teaching hospital & postnatal clinic.	Self-reported, using English or local-version EPDS.	94.0 % of the participants	NM	NM	Psychiatrist & psychiatric nurse	Diagnostic interview using ICD −10 for mental disorders	8/9^b^	75	97	75	97
Adewuya et al., (2006) [31]	182 pregnant women (≥32 weeks) conveniently recruited from the antenatal clinics of 5 health centres, located in a semi-urban town of western Nigeria (15.4 % were illiterate).	Interviewer administered for illiterate women.	86 (all 75 scored ≥ 6 & rest 10 % randomly selected out of those scoring <6)	NM	Yes	2 psychiatrists	MINI DSM-IV of Depression	9/10	86.7	91.5	68	97
Pakistan												
Rahman et al., (2005) [39]	541/570 postnatal women (10 to12 weeks) recruited from a rural community of the Rawalpindi sub-district. About 75 % were illiterate.	Interviewers	All 541	Yes	Yes	2 Mental health professionals	SCAN for ICD-10 for depressive disorders *(translated & adapted)*	9/10^b^	81.5	73.1	52.6	NM
Husain et al., (2013) [26]	601/664 postnatal women (0–36 months) recruited from an urban slum in the capital Karachi.		All 601	Yes	Yes	NM	CIS-R, ICD −10^b^ for depression	13/14^b^	79	74	82	70
Mongolia												
Pollock et al., (2006) [32]	94/100 women (in reproductive age) conveniently recruited from two specialised psychiatric units (55) & rest from the 3 community based immu1nization clinics in the capital Ulaanbaatar (adult literacy rate near 100 %)	Not clear	All 94	Yes	NM	1 bstetrician-an/gynaecologist, 1 psychologist	CIS-R, ICD-10 for depressive disorders *(translated & adapted)*	12/13^b^	80.9	61.7	67.9	76.3
Bangladesh												
Gausia et al., (2007) [25]	100/126 postnatal women \(6–8 weeks) conveniently recruited from a child immunization clinic in Dhaka, 11 % were illiterate.	1 interviewer	All 100	Yes	Yes	1 psychiatrist	SCID DSM-IV of depression	9/10	88.9	86.8	40	98.6
Sri Lanka									Pregnant			
Rowel et al., (2008) [37]	465 perinatal women conveniently recruited for this study: of them 265 were pregnant (≥34 weeks) and attending antenatal clinics. The other 204 were postpartum women (≥6 weeks) attending a family planning or child wellbeing clinic (all could read & write).	Not clear	All 465	NM	NM	1 psychiatrist	Diagnostic interview using ICD-10 for mental disorders	8/9	90.7	86.8	NM	NM
									Postnatal			
									89.9	78.9	NM	NM
Ethiopia												
Hanlon et al., (2008) [33]	101 postnatal women (median 5 Months) recruited from the Butajra sub-district (rural community)	20 Interviewers	52 participants	NM	NM	Psychiatrists	CPRS DSM-IV of common mental disorders	5/6	76.5	36.1	NM	NM
Tesfaye et al., (2010) [35]	100/102 postnatal women (6 to 14 weeks) conveniently recruited from child immunization and/or postnatal clinics in 2 primary health care centres, located in the peri-urban area of the capital Addis Ababa. 21 % were illiterate.	Interviewers	All 100	Yes	Yes	2 psychiatrists		6/7	78.9	75.3	42.9	93.8
Ghana												
Weobong et al., (2009) [38]	160 pregnant women (5–11 week) identified from the database of 1/6 districts where vitamin A trial was implemented.	Interviewers	About half	Yes	Yes	1 psychologist	SCAN for common mental disorders	10/11	78	73	22	97
Zimbabwe												
Chibanda et al., (2010) [36]	210/223 postnatal women (6–7 weeks) conveniently recruited from the 2 primary health care centres, located in a peri-urban area of the capital Harare (74 % completed secondary education).	6 interviewers	All 210	Yes	Yes	2 psychiatrists	DSM-IV of major depression	10/11^b^	88	87	74	94
Vietnam												
Tran et al., (2011) [27]	364/392 perinatal women (199 were ≥ 28 weeks pregnant & rest were 4–6 weeks postpartum) from randomly selected commune health centres in the capital Hanoi. Rural women were recruitedfrom the Ha Nam province.	Interviewers	All 364	Yes	Yes	1 psychiatrist	SCID DSM-IV of depression, generalised anxiety, panic disorders	3/4^b^	69.7	72.9	69.7	72
Malawi												
Stewart et al., (2013) [34]	224 pregnant women (2nd trimester) conveniently recruited from a rural district hospital. Only Chichewa speakers were recruited for this study.	2 interviewers	92 (all scored ≥9; every other for those scored 6–9 & every fourth scored ≤5)	NM	Yes	1 (NM)	SCID DSM-IV of depressive disorders	4/5^b^	68.7	88.2	35.8	97.4

^a^While the validation of a Nepalese version EPDS was published by Nepal et al., [24], it is not clear that particular Nepalese version was used by Regmi et al. [40];

*NM* not mentioned: ^b^of the multiple cut-offs, presented one that had Sensitivity and Specificity nearest to 80 %;

^c^Diagnostic Instrument: *SCID* Structured Clinical Interview for DSM, *CIS-R* Revised Clinical Interview Schedule, *MINIPLUS* Mini International Neuropsychiatric Interview, *SCAN* Schedule for Assessment in Neuropsychiatry, *CPRS* Comprehensive Psychopathological Rating scale; ^d^Diagnostic criteria: *ICD10* WHO International Classification of Disease, *DSM-IV* Diagnostic and Statistical Manual for Mental Disorders- Fourth Edition

^e^ *Se* Sensitivity, ^f^ *Sp* Specificity, ^g^ *PPV* Positive predictive value, ^h^ *NPV* Negative predictive value

Generation of data on performance of these 14 LLV-EPDS was mainly carried out by recruiting study participants from ante- or postnatal or immunisation clinics (13/17 studies) [24, 25, 28–32, 34, 36–38, 40]. More than half of the studies (10/17) recruited women who had recently given birth (0 to 36 months ago) [24–26, 28, 30, 33, 35, 36, 39, 40]; while 4/17 studies included women who were currently pregnant [29, 31, 34, 38]; there were both pregnant women and those who had recently given birth in two studies [27, 37] and in one study all participants were women of reproductive age (18–40 years) [32]. Of the 11 publications [24, 25, 27–29, 31, 32, 34–36, 39] that mentioned participants’ literacy status, almost all had a predominance of literate participants (67 to 89 %), except in a rural community-based investigation from Pakistan, in which only a quarter of women could read [39]. In 10/17 studies, interviewers administered the LLV-EPDS [25, 26, 28, 29, 31, 33, 35, 36, 38, 39].

Diagnostic interviews were carried out to establish clinical cut-off points. Of the 17 studies, in 11studies all participants who were screened using LLV-EPDS were also recruited for diagnostic interviews. In three studies, all those scoring higher than certain scores (≥6, ≥9 and ≥13) and a randomly selected sample of woman who scored below the cut-offs were included. In the remaining two studies, about half the participants were included (Table 4).

Psychiatrists or psychologists carried out the diagnostic interviews in 12/17 studies, but in eight of these publications, it is not clear if they were blinded to the initial screening results [24, 28–30, 32, 33, 37, 40]. In addition, in eight studies [24, 28–31, 33, 37, 40] it is not clear whether the diagnostic interviews were carried out on the same day as the screening. Furthermore, although interviews were conducted in the local language, the standard diagnostic protocol (SDP) were translated into respective local languages in only three studies [29, 32, 39], while only two of the SDP had been culturally adapted [32, 39]. In 6/17 studies, there was more than one diagnostic interviewer; but only one of these investigated inter-rater reliability and reported excellent reliability between psychiatrists (kappa = 0.82) [33].

### Psychometric Properties

The 17 studies of the 14 LLV-EPDS revealed wide variation in their psychometric properties. The range of cut-off scores selected for detecting any common mental disorder was 3/4 to 11/12; and where specific conditions were listed, the range of cut-off scores was 3/4 [27] to 13/14 [26] for depression (mild and moderate) and 4/5 to 12/13 for severe depression [40]. The scores for sensitivity (identifying true cases) ranged from 69.7 % [27] up to 100 % [29, 40]. The scores for specificity (identifying those without PCMDs) ranged from 36.1 % [33] up to 97 % [30]. The positive predictive value (PPV, which is the proportion of respondents who scoring positive in the screening, who were confirmed to have a common mental disorder by clinical interview) ranged from 22 % [38] up to 82 % [26]; the negative predictive value (NPV, which is the proportion of the respondents scoring negative in the screening who were confirmed as having no mental disorders) ranged from 70 % [26] to 100 % [40]. The range of cut-off points for detecting depression among pregnant women was slightly lower 4/5 [34] to 12/13 [29] than among women who had recently given birth 5/6 [33] to 13/14 [26].

## Discussion

To our knowledge, this systematic review is the first assessment of EPDS versions translated and adapted for use in low- and lower-middle-income countries (LALMICs). Additionally, this is the first study to use a new set of process-based criteria (Fig. 1) to assess the reliability and validity of LLV-EPDS comprehensively. We acknowledge the possible limitation of this review, that some studies on LLV-EPDS may have been missed, if they had been published in languages other than English in non-indexed journals. However, we think this is unlikely, because we followed rigorous strategies to identify pertinent studies published in non-indexed journals.

We found that of the 82 countries classified by the World Bank (in 2015) as LALMICs, the Edinburgh Postnatal Depression Scale (EPDS) had been formally validated in 12 (14.6 %) countries in 14 native languages. We found psychometric properties of these 14 LLV-EPDS from low-and middle-income countries were lower than that for the original English EPDS [6]. Our finding is consistent with findings from earlier three systematic reviews that included studies from high-, middle- and low-income countries [3, 4] and African countries [12].

A central finding of our systematic review is that utility of these 14 local versions EPDS for screening PCMDs is questionable, as none met the recommended validation standard of ≥80 % in the three key parameters: sensitivity, specificity and positive predictive value [41]. The process-based appraisal indicated that lower psychometric property of these LLV-EPDS might be related to compromises made during translation, cultural adaptation and empirical validation (Tables 3 and 4). The psychometric properties of the LLV-EPDS were found to be better when the process-based criteria were followed. In Ethiopia, Tesfaye et al. [35] achieved an almost two fold improvement in specificity (75.3 % vs. 36.1 %), by inclusion of more suitable local expressions than in the earlier Amharic version EPDS [33]. The Bangla EPDS, was the only one which met all steps and criteria for culturally sensitive translation, cultural adaptation and empirical validation and was the one study that demonstrated high sensitivity (88.9 %) and specificity (86.8 %) [19] (Table 4).

Sub–optimal sensitivity and specificity in these LLV-EPDS might also have been attributable to the recruitment of women who did not represent the wider perinatal population during empirical validation (Fig. 1, criteria: 15–21). For instance, more than half of the studies (10/17) recruited participants from health facilities, most usually a single, urban health institution, using convenience sampling. This is especially problematic in settings where many women give birth in primary care facilities or at home. Selection bias is apparent as there was a predominance of well educated women in these study samples. While they might understand direct, literally translated LLV-EPDS, it is much less likely that women of low literacy or education will understand them. The link between non-random selection of participants from health institutions and a poorly translated LLV-EPDS is shown by the study from Nigeria. In that country, while about half of the female population (50.6 %) are illiterate [42], more than two thirds of the study participants who were recruited from antenatal clinics were highly educated (bankers, teachers/lecturers, big business owners and civil servants). In this study, the LLV-EPDS had high sensitivity (86.7 %) and specificity (91.5 %), even though it didn’t meet most of our recommended steps for culturally sensitive translation processes [31]. This suggests that these highly educated participants having greater emotional literacy [3, 23] and familiarity with test-taking [7], but does not provide evidence that this LLV-EPDS will be useful for the majority who have not had opportunities for education and social participation.

Further areas of inconsistency and suboptimal practice appeared to have occurred during the process of formal validation against a diagnostic interview (Fig. 1, criteria: 22–28). In 11/17 studies the interviews were carried out by psychiatrists or psychologists. However, in 8 of these studies, diagnosis might have been influenced by changes in the interviewees’ psychological state as the screening by LLV-EPDS and diagnostic interviews were not conducted on the same day [24, 28, 30, 31, 33, 34, 37, 40]. Further, the diagnosis might have been biased by the psychiatrists or psychologists being aware of the screening results, as only 8 studies reported that they were blinded to the initial screening results. Moreover, even though interviews were carried out in local languages, the accuracy of both screening and diagnosis may be influenced by participants’ limited understanding of English colloquialisms. Only four studies made amendments to the LLV-EPDS so that it was suitable for administration to participants with low literacy, and tried to attain semantic equivalence to the local context by using local expressions [19, 23, 33, 35]. For instance, the statement in the original EPDS “Life is getting on top of me” is intended to detect an experience in which a woman feels that the demands imposed on her, exceed her capacity to manage them. However, it was interpreted by some women in Vietnam as meaning literally that things were being placed on top of them as might occur during a flood or other natural disaster [23]. Although all diagnostic interviews were presumed to be conducted in local languages, only two reported that standardised diagnostic protocols had been culturally adapted. It is possible that in the other studies participants might have not understood and or mis-understood questions asked by clinicians [32, 39].

Conceptual equivalence was generally not established between the LLV-EPDS and the original English EPDS [10]. In half of these studies, the back-translations to English might have been influenced by knowledge of the original version, as both forward and back-translation were carried out by the same panels. Additionally, the back-translated English version was reviewed by native-English-speakers in only one study [19]. Conceptual and operational disparities between the original English version and the LLV-EPDS were not investigated and tests of equivalence were not performed.

There is considerable variation in cut-off points to detect clinically significant symptoms among the 14 LLV-EPDS from non-English speaking low- and lower-middle-income countries. In general lower cut-off scores compared to the English version were found. This probably reflects differences in cultural norms about emotional expression and emotional literacy [7, 9, 23]. The EPDS, developed in Britain, reflects the psychiatric paradigm that experiences of low mood are episodic and represent change from a usual state. It is inaccurate to presume that this is a universal situation. In resource constrained settings, where many women experience chronic social and economic adversity it is probable that they might not experience change from a usual state, but rather could be chronically distressed, so answers to questions about change would be negative [23]. There is also potential confusion, and perhaps linguistic limitations which mean that subtle emotional distinctions, for example being anxious or being scared might not be available or in widespread use and therefore lead to responses that do not reflect reality.

It would appear overall that formal validation of the EPDS following proposed process-based criteria is more likely to derive precise cut-off points appropriate to the local setting and /or population. Having a LLV-EPDS with an imprecise cut-off point has potentially serious implications. On one hand, an inaccurately high cut-off point imported from a high-income Anglophone setting might lead to under-detection of women with PCMDs. This means women’s needs might go unrecognised and unassisted and lead to under-estimation of PCMDs burden for a particular country or population. On the other hand, if a cut-off point is too low, women might be classified as having a clinically significant condition of mental disorders which may lead to unnecessary treatment and potentially, stigma and discrimination, in particular in societies where experiences of human suffering are poorly understood. Inaccurate classification of ‘cases’ may further strain the already over-burdened health systems of low- and lower-middle-income countries [11].

## Conclusions

**Fig. 3:**
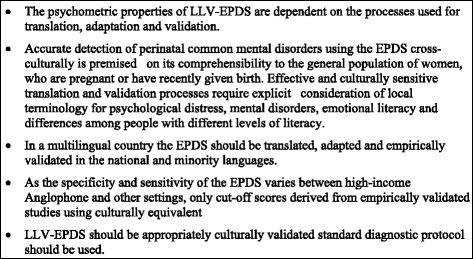
Recommendations for optimising psychometric properties of the LLV-EPDS in low- and lower-middle-income countries

It is commendable that researchers and clinicians in several resource-constrained countries have made great efforts to improve early detection and timely management of PCMDs. However, this review indicated that currently available local language versions of the Edinburgh Postnatal Depression scales (LLV-EPDS) from low-and lower- middle income countries are of some value, but most of them had deficiencies in translation, cultural adaptation and validation processes. Screening instruments with poor psychometric properties might have far-reaching implications for clinical practice, public policy and research. We recommend a systematic approach to the translation, cultural adaptation and empirical validation of local language versions of the EPDS that adheres to the steps outlined in Fig. 3. This approach will facilitate the development of more precise and validated screening tools for detection and management of PCMDs among women in resource constrained settings.

## Additional files

Additional file 1: Ten items of the Edinburgh Postnatal Depression Scale [5]. (DOCX 15 kb) Additional file 2: PRISMA (2009) checklist for studies included for this systematic review. (DOCX 25 kb) Additional file 3: Literature search strategy for systematic review on the reliability and validity of the EPDS in low and lower-middle income countries (LALMICs). (DOCX 14 kb) Additional file 4: Definitions of the Process-based criteria. (DOCX 20 kb)
