# Supplementary material for: Reliability and validity of the Edinburgh Postnatal Depression Scale (EPDS) for detecting perinatal common mental disorders (PCMDs) among women in low-and lower-middle-income countries: a systematic review
Source: BMC Pregnancy Childbirth. 2016 Apr 4;16:72. doi: 10.1186/s12884-016-0859-2 (PMC4820998; doi:10.1186/s12884-016-0859-2)
Supplement: Additional file 1: — Ten items of the Edinburgh Postnatal Depression Scale [5]. (DOCX 15 kb) [file 12884_2016_859_MOESM1_ESM.docx]

Additional file 1: Ten items of the Edinburgh Postnatal Depression Scale [5]

______________________________________________________________________________

1. I have been able to laugh and see the funny side of the things

a. As much as I always could

b. Not quite so much now

c. definitely not so much now

d. Not at all

2. I have looked forward with enjoyments to things-

a. As much as I ever did

b. Rather less than I used to

c. Definitely less than I used to

d. Hardly at all

3. I have blamed unnecessarily to myself when things went wrong

a. Yes, most of the time

b. Yes, some of the time

c. Not very often

d. No, never

4. I have been anxious or worried for no good reasons

a. No, not at all

b. Hardly, ever

c. Yes, sometime

d. Yes, very often

5. I have felt panicky or scared for no good reasons

a. Yes, quite a lot

b. Yes, sometimes

c. No, not much

d. No, not at all

6. Things have been getting on top of me

a. Yes, most of the time I haven’t been able to cope at all

b. Yes, sometimes I haven’t been able to cope as usual

c. No, most of the time I have coped quite well

d. No, I have been coping as well as ever

7. I have been so unhappy that I had difficulty in sleeping

a. Yes, most of the time

b. Yes, some of the time

c. Not very often

d. No, never

8. I have felt sad or miserable

a. Yes, most of the time

b. Yes, some of the time

c. Not very often

d. No, never

9. I have been so unhappy that I have been crying

a. Yes, most of the time

b. Yes, some of the time

c. Not very often

d. No, never

10. The thought of harming myself has occurred to me

a. Yes, quite often

b. Sometimes

c. Hardly ever

d. Never

_________________________________________________________________________________
