# Supplementary material for: Reliability and validity of the Edinburgh Postnatal Depression Scale (EPDS) for detecting perinatal common mental disorders (PCMDs) among women in low-and lower-middle-income countries: a systematic review
Source: BMC Pregnancy Childbirth. 2016 Apr 4;16:72. doi: 10.1186/s12884-016-0859-2 (PMC4820998; doi:10.1186/s12884-016-0859-2)
Supplement: Additional file 2: — PRISMA (2009) checklist for studies included for this systematic review. (DOCX 25 kb) [file 12884_2016_859_MOESM2_ESM.docx]

Additional file 2: PRISMA (2009) checklist for studies included for this systematic review

| Section /Topic | # | Checklist item | Reported Page # |
| --- | --- | --- | --- |
| TILE | | | |
| Title | 1 | Identify the report as a systematic review, meta-analysis, or both. | Page 1 |
| ABSTRACT | | | |
| Abstract | 2 | Provide a structured summary including, as applicable: background; objectives; data sources; study eligibility criteria, participants, and interventions; study appraisal and synthesis methods; results; limitations; conclusions and implications of key findings; systematic review registration number. | Page 2 |
| INTRODUCTION | | | |
| Rational | 3 | Describe the rationale for the review in the context of what is already known | Page 4-5 |
| Objectives | 4 | Provide an explicit statement of questions being addressed with reference to participants, interventions, comparisons, outcomes, and study design (PICOS). | Page 5 |
| METHOD | | | |
| Protocol and Registration | 5 | Indicate if a review protocol exists, if and where it can be accessed (e.g., Web address), and, if available, provide registration information including registration number. | For this systematic review study, we developed a new set of Process-based criteria for appraising formally validated local language versions EPDS (LLV-EPDS). That could be taken as the Review Protocol, which is provided with our manuscript as Figure 1. |
| Eligibility criteria | 6 | Specify study characteristics (e.g., PICOS, length of follow-up) and report characteristics (e.g., years considered, language, publication status) used as criteria for eligibility, giving rationale. | Page 6 |
| Information source | 7 | Describe all information sources (e.g., databases with dates of coverage, contact with study authors to identify additional studies) in the search and date last searched. | Page 6 |
| Search | 8 | Present full electronic search strategy for at least one database, including any limits used, such that it could be repeated. | 6 and Box 2. |
| Study selection | 9 | State the process for selecting studies (i.e., screening, eligibility, included in systematic review, and, if applicable, included in the meta-analysis). | Page 6-7 |
| Data collection process | 10 | Describe method of data extraction from reports (e.g., piloted forms, independently, in duplicate) and any processes for obtaining and confirming data from investigators. | Page 8 (Inserted and described in the 2^nd^ revised version) |
| Data items | 11 | List and define all variables for which data were sought (e.g., PICOS, funding sources) and any assumptions and simplifications made. | Page 8, and the list of variables, for which data were sought, is provided in the Figure 1 and defined in Box 3. |
| Risk of bias in individual studies | 12 | Describe methods used for assessing risk of bias of individual studies (including specification of whether this was done at the study or outcome level), and how this information is to be used in any data synthesis. | We incorporated variables which potentially compromise reliability and validity of local language version EPDS in the new-set of process-based criteria (Figure 1) and Page 8. |
| Summary Measures | 13 | State the principal summary measures (e.g., risk ratio, difference in means). | For this study principal summary measures were five Psychometric properties of the local language version EPDS. These mentioned in the  Figure 1, Page: 3-5, 14-15, 19-21. Table 1, 4 |
| Synthesis of results | 14 | Describe the methods of handling data and combining results of studies, if done, including measures of consistency (e.g., I2) for each meta-analysis. | 8  (We presented /data on adherence to process-based criteria by narrative summaries. Objective of this study is identifying modifiable reasons behind lower validity formally validated LLV-EPDS. So, we didn’t conducted meta-analysis in our study). |
| Risk of biases across studies | 15 | Specify any assessment of risk of bias that may affect the cumulative evidence (e.g., publication bias, selective reporting within studies). | Approach used to address related to publication mentioned in the Method section (Page 6-7) and Discussions section (Page 15).  Selective reporting within studies, were critically reviewed using the new process-based criteria, we specifically developed for this study. |
| Additional analyses | 16 | Describe methods of additional analyses (e.g., sensitivity or subgroup analyses, meta-regression), if done, indicating which were pre-specified. | Assessment of LLV-EPDS’s developmental process using the process-based criteria could be taken as an additional analysis. |
| RESULTS | | | |
| Study selection | 17 | Give numbers of studies screened, assessed for eligibility, and included in the review, with reasons for exclusions at each stage, ideally with a flow diagram. | Page 8-9, and Figure 2 |
| Study characteristics | 18 | For each study, present characteristics for which data were extracted (e.g., study size, PICOS, follow-up period) and provide the citations | Page 9 |
| Risk of bias within studies | 19 | Present data on risk of bias of each study and, if available, any outcome level assessment (see item 12). | Page 8-15  Table 1- 4 |
| Results of individual studies | 20 | For all outcomes considered (benefits or harms), present, for each study: (a) simple summary data for each intervention group (b) effect estimates and confidence intervals, ideally with a forest plot. | Table 1- 4 |
| Synthesis of results | 21 | Present results of each meta-analysis done, including confidence intervals and measures of consistency. | In page 8-15, narrative summary of evidence presented). |
| Risk of bias across studies | 22 | Present results of any assessment of risk of bias across studies (see Item 15). | We presented risk of bias across studies as narrative of adherence to the process-based criteria. Since, the process-based criteria are specifically developed for this study. It could be taken as additional analysis. |
| Additional analysis | 23 | Give results of additional analyses, if done (e.g., sensitivity or subgroup analyses, meta-regression [see Item 16]). |  |
| DISCUSSION | | | |
| Summary evidence | 24 | Summarize the main findings including the strength of evidence for each main outcome; consider their relevance to key groups (e.g., healthcare providers, users, and policy makers). | Page 15 and 21 |
| Limitations | 25 | Discuss limitations at study and outcome level (e.g., risk of bias), and at review-level (e.g., incomplete retrieval of identified research, reporting bias). | Page 15 |
| Conclusions | 26 | Provide a general interpretation of the results in the context of other evidence, and implications for future research | Page 21-22 |
| Funding | 27 | Describe sources of funding for the systematic review and other support (e.g., supply of data); role of funders for the systematic review. | Page 23 |
