# Supplementary material for: Reliability and validity of the Edinburgh Postnatal Depression Scale (EPDS) for detecting perinatal common mental disorders (PCMDs) among women in low-and lower-middle-income countries: a systematic review
Source: BMC Pregnancy Childbirth. 2016 Apr 4;16:72. doi: 10.1186/s12884-016-0859-2 (PMC4820998; doi:10.1186/s12884-016-0859-2)
Supplement: Additional file 3: — Literature search strategy for systematic review on the reliability and validity of the EPDS in low and lower-middle income countries (LALMICs). (DOCX 14 kb) [file 12884_2016_859_MOESM3_ESM.docx]

| Additional file 3: Literature search strategy for systematic review on the reliability and validity of the EPDS in low and lower-middle income countries (LALMICs)  1."Translation or Cultural adaptation or Validation"  2. "Edinburgh Postnatal/Postpartum Depression Scale or EPDS"  3. "prenatal or antenatal or pregnancy or postnatal or postpartum"  Combined terms: 1 AND 2 AND 3  *PUBMED:  "translation, adaptation and validation of Edinburgh postnatal/postpartum depression scale or EPDS" |
| --- |
