# Supplementary material for: Reliability and validity of the Edinburgh Postnatal Depression Scale (EPDS) for detecting perinatal common mental disorders (PCMDs) among women in low-and lower-middle-income countries: a systematic review
Source: BMC Pregnancy Childbirth. 2016 Apr 4;16:72. doi: 10.1186/s12884-016-0859-2 (PMC4820998; doi:10.1186/s12884-016-0859-2)
Supplement: Additional file 4: — Definitions of the Process-based criteria. (DOCX 20 kb) [file 12884_2016_859_MOESM4_ESM.docx]

Additional file 4: Definitions of the Process-based criteria:

Step 1: Forward translation is translation of the original English version EPDS into local language version EPDS (LLV-EPDS) [10].

Criterion 1: Number of translators in the forward-translation panel should be more than one.

Criterion 2: Professional background refers to forward translators should being native-language-speakers, having fluency in English language and

had prior experiences of translating psychometric instruments.

Step 2: Backward translation is translation of the LLV-EPDS back into English language [10].

Criterion 3: Provision of a different translation panel for backward-translations.

Criterion 4: Number of participants in the backward translation panel should be more than one.

Criterion 5: Professional background refers to the backward-translators should being fluent in both local language and the English language.

Additionally, they must not have knowledge of the original EPDS prior and/or during the translation process.

Criterion 6: Revision of the backward translated version by native-English-speakers (aimed for conceptual equivalence with original English version).

Step 3: Resolution of difficulties and differences in translations for addressing problematic terms and items translation [18, 19, 21].

Criterion 7: Convening discussions in a committee.

Criterion 8: Number of participants in the committee.

Criterion 9: Participants background refers to expertise of those attended in the discussions (recommended inclusion of multidisciplinary health

professionals along with lay-persons).

Step 4: Pretesting is assessment of the LLV-EPDS among a small group of women (who were pregnant or had recently given birth) representative to the

Study population.

Criterion 10: Probing refers to assessment for comprehension of the LLV-EPDS.

Step 5: Amendments is based on pretest-findings modifications of preliminary LLV-EPDS for improving its understanding & comprehension by the

targeted study population.

Criterion 11: In Format is altering pattern of questioning & response statements to suit its administration to women with no/low literacy.

Criterion 12: In Semantic is inclusion of appropriate local expressions.

Step 6: Tests of equivalence is statistical assessments for establishing cultural sensitivity of the LLV-EPDS [16, 19].

Criterion 13: Conceptual equivalence is correlation of scores between the LLV-EPDS and the original English version EPDS.

Criterion 14: Operational equivalence is correlation of scores between self-administrated and interview administered LLV-EPDS.

Step 7: Generating data on performance of the LLV-EPDS (Screening instrument) is administration of the LLV-EPDS to women (who were pregnant or

had recently given birth) representative to the study population [1, 7, 11, 21, 22]

Criterion 15: Study design refers to study type used.

Criterion 16: Sample size refers to number of participants recruited, which should be determined using standard formula to achieve adequate power.

Criterion 17: Child-bearing stage refers to recruited samples whether were in reproductive age or were pregnant or recently had given birth.

Criterion 18: Literacy status refers to participants' being literate or illiterate.

Criterion19: Place of residence refers to participants' living in rural or urban area.

Criterion 20: Mode of administration refers LLV-EPDS administration, which is self-administration by participants' themselves or by interviewer (s).

Criterion 21: Test of inter-rater reliability between interviewers is assessment of one participant's responses by two observers.

Step 8: Conforming LLV-EPDS outcomes by the Diagnostic interview is ascertaining initial screening outcomes (by LLV-EPDS) by mental health

professional [3, 7, 22].

Criterion 22: Number of interviewees recruited for the diagnostic interview refers whether all interviewees or partially (those selected on

the basis of scores in the screening) were interviewed for ascertaining screening outcomes.

Criterion 23: Time-gap between the screening (by the LLV-EPDS) and the Diagnostic interview refers to time-lapse between these two assessments

(recommended less than 24 hours to prevent bias due to changes in study-participants’ mood).

Criterion 24: Diagnostic interviewer’(s) professional background refers to their field of expertise.

Criterion 25: Diagnostic interviewer blinded of the screening result refers preventing them having knowledge of the initial screening outcomes.

Criterion 26: Standard Diagnostic Protocol refers to specific instrument used for the diagnostic interviews.

Criterion 27: Cultural sensitivity of the Standard Diagnostic Protocol refers to whether it was translated and culturally adapted.

Criterion 28: Test of reliability between diagnostic interviewers refers assessment of inter-rater differences in ratings, which is measured by kappa value.

Step 9: Deriving parameters of validity refers to extrapolation of following 5 psychometric properties of a given LLV-EPDS [17].

Criterion 29: Cut-off point is the clinical score for ascribing those screened as case or non-case.

Criterion 30: Sensitivity is the proportion of respondents identified as true cases by LLV-EPDS (%).

Criterion 31: Specificity is the proportion of respondents identified as false positive case by LLV-EPDS (%).

Criterion 32: Positive predictive value is the proportion of respondents, who scored positive in the screening (by LLV-EPDS) also confirmed to have

Mental disorder in the diagnostic interview (%).

Criterion 33: Negative predictive value is the proportion of the respondents, who scored negative in the screening (by LLV-EPDS), also confirmed of not

having mental disorders in the diagnostic interview (%).
